# An approach for developing integrated undergraduate medical curriculum

**DOI:** 10.12669/pjms.344.14565

**Published:** 2018

**Authors:** Ashfaq Akram, Farzana Rizwan, Kamran Sattar, Jalal Ibrahim S Hadi, Sultan Ayoub Meo

**Affiliations:** 1 Ashfaq Akram, Medical Education, Riphah International University, Islamabad, Pakistan. College of Medicine, King Saud University, Riyadh, Saudi Arabia; 2 Farzana Rizwan, Faculty of Medicine, Taylor University, Kuala Lumpur, Malaysia; 3 Kamran Sattar, Medical Education, College of Medicine, King Saud University, Riyadh, Saudi Arabia; 4 Jalal Ibrahim S Hadi, International Medical School, Management and Science University-Shah Alam, Malaysia; 5 Sultan Ayoub Meo, Department of Physiology, College of Medicine, King Saud University, Riyadh, Saudi Arabia

**Keywords:** Basic sciences, Clinical science, Curriculum, Integration, Module, Undergraduate

## Abstract

**Background and Objectives:**

Medical schools are to develop integrated medical curricula because the term ‘integrated curriculum’ has grown up and flourished globally and it has become mandatory to align the medical education with the global concept in Pakistan. This paper aims to present a guideline to design an undergraduate integrated medical curriculum.

**Methods:**

Various themes are used to develop integrated curriculum which are basic medical science, simulation skills, clinical science, personality development, research, entrepreneurship and pre specialization. Each theme is subdivided, termed a module and its contents primarily focus on particular aspect.

**Results:**

Knowledge, skill and attitude, embodied in themes or modules, are planted in specific way that they have horizontal as well as vertical integration. There is no boundary of various traditional disciplines in template of five years curriculum. For example, diagnosis is a theme which carries contents from medicine, surgery, orthopedics etc.

**Conclusion:**

The blueprint introduced in this paper would help medical educators to draft integrated medical curricula for those institutions which intend to switch their medical programs from traditional to integrated one.

## INTRODUCTION

Traditional medical curricula give priority to large volumes of clinical work and memorized knowledge.^1^ The undergraduate medical curriculum ‘2+3’ has been followed over many decades in Pakistan and encompasses basic medical sciences (anatomy, physiology, pathology, pharmacology etc.) followed by clinical sciences (medicine, surgery, pediatrics etc.). Basic science knowledge is taught in first two years while clinical skills are provided over a period of three years. In fact, a barrier between basic and clinical sciences exists. A major disadvantage of this type of curriculum format is that the learners forget the basic science knowledge when they enter into clinical phase, thus, it is viewed as inadequate curriculum to prepare the future physicians for twenty first century.^2^,^3^ Globally, the bar between basic and clinical sciences has been broken down to develop concepts of knowledge with clinical application which promotes retention of knowledge and acquisition of repetitive skills.^4^ There is demand that beyond a thorough understanding of applied anatomy and pathophysiology, medical graduates should possess knowledge across the basic and applied sciences, be experts in different areas of medical skills and have exemplary attitude.^5^

Harden et al.,^6^ described the integrated medical curriculum. The term was supported by many medical education organizations such as the Association of American Medical Colleges^7^, the General Medical Council, UK^8^ and the Australian Medical Council.^9^ An integrated curriculum establishes significant linkages between the subjects or skills that are mainly addressing various subject areas to improve the learning experiences. Moreover, it allows opportunities for all the stakeholders to think outside the box. Pakistani medical institutions intend to develop integrated medical curricula in order to harmonize with the global concept in medical education. We are presenting a concept (approach) to develop undergraduate integrated medical curriculum.

## METHODS

The distinct and manifested medical themes are deployed for integrated undergraduate medical curriculum. Basic medical science, medical simulation, clinical science, personal and professional development, research, entrepreneurship, and pre-specialization are the key ones and contents of each one are scrutinized and further divided into subcategories termed as modules.

**1. Basic Medical Science** theme correlates normal body structures to abnormal structures, disease mechanisms and drug interactions coherently, logically and enables the students to understand illness mechanisms and pharmacotherapy concepts. This theme is further categorized into ‘basic foundation, blood, respiratory, gastro-intestinal tract, cardiovascular, renal, endocrine, reproductive and nervous modules. The term ‘module’ could also be replaced by ‘block’ and each module or block should also be given different name or two modules suggested hereby may be combined based on the included contents as determined by medical schools independently.

**2. Medical Simulation** theme is about to acquire clinical skills in safe environment. The skills required in hospital practice are exercised on simulated patients and provides risk free, efficient and competency to medical gradautes.^10^ The gowning and gloves, handling instruments, knot tying and suturing are examples of surgical skills while cardiac murmurs, palpating liver, spleen, carotid bruit, heart rate funduscopy and apex beat location are medical based skills.^11^

**3. Clinical Science** theme is subdivided into ‘disease, diagnosis and treatment’ modules. ***Disease module*** emphasizes on mechanisms, signs and symptoms of different diseases. The pathophysiology, aetiology and laboratory investigations of various illnesses are overspread to correlate the basic fundamental knowledge of human body and disease process. Thus it should be divided into three modules a: mechanism of disease (Disease I); b: illness associated with surgical disciplines (Disease II) and c: sickness related to medical disciplines (Disease III).

***Diagnosis module*** combines the collective strengths of fundamental problems of various medical disciplines and creates strong foundation in medical students and they are able to differentiate common medical problems. It is further divided into two or more than two phases and each phase progresses with advanced and complex index. One phase termed as Diagnosis I should include medical based disciplines (medicine, pediatrics, primary care, psychiatry etc.) while diagnosis of surgical based disciplines (surgery, obstetrics and gynecology, otolaryngology, orthopaedics etc.) should be taught in Diagnosis II module. Diagnosis I & II may be given specific names by medical schools.

***Treatment module*** focus on indications of various aspects of medical and surgical treatment protocols and is segmented such as special senses, mother and baby, trauma, oncology etc. Furthermore, it emphasizes on treatment of emergency problems, geriatric care, and common public health issues. This module may be divided into Treatment I to IV highlighting the specific guidelines of health care for contents to be taught in diagnosis theme.

**4. Personal and Professional Development (PPD)** theme probes attitude and moral aspects of medical graduates along with knowledge, skills and belief in relation to working in medical environment. This theme is designated with different titles rather than PPD I, II, III and IV. The term PPD or similar titles are used in various medical curricula to enhance communication, leadership, team work and ethical values.

**5. Research** theme is added in undergraduate integrated medical curriculum to provide a strong foundation with research knowledge, skills and attitudes (team work, communication, critical thinking etc). Moreover, they are trained in understanding the current medical literature and research methodology. Therefore, it should be divided into Research methodology I and II. Basic information about research methods and research projects including biostatistics are given in Research I and II modules respectively.

**6. Entrepreneurship** theme is to be included in integrated curriculum. Traditionally, it has never been considered for medical students. The aim of this theme is to make medical students aware of business concepts. The students are equipped with application of clinical practice, venue selection and requisite human workforce for business setup. It was observed that knowledge related to medical business and finance had a positive impact and value on students’ perception towards business planning.^12^

**7. Pre-specialization:** The goal of the Pre-specialization theme is to direct and shape the mindset of students for postgraduate studies of their choice. This module furnishes the intentions of undergraduate medical students in order to achieve more knowledge and comprehensive skills in a higher resourced environment.^13^

## RESULTS

### Horizontal and vertical integration of themes

**Fig.1:**
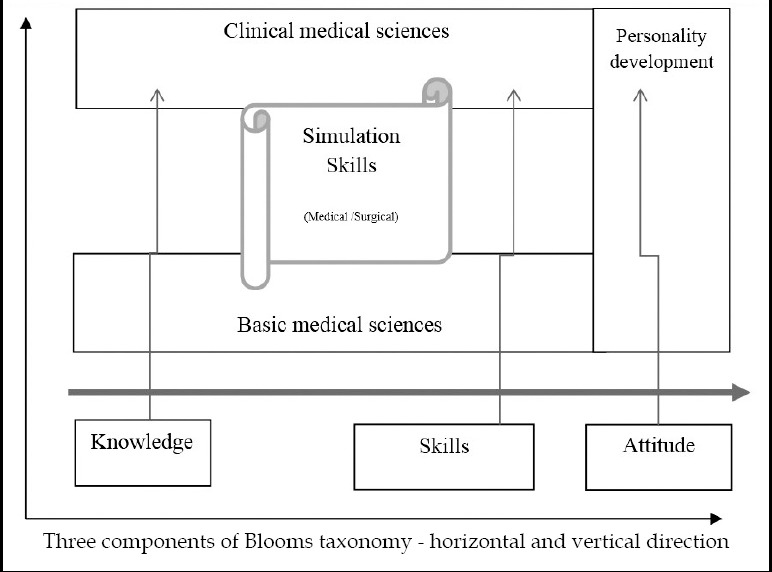
Integration of basic and clinical sciences

The seven themes are presented in Table-I in alignment of the bloom’s taxonomy. There is horizontal as well as vertical integration (Fig.1). Clinical skills are chained with basic medical sciences through simulated skills in arranged topics, thus cognitive and psychomotor domains are combined while affective domain is placed vertically. However, debate on detailed description of topics and their placement in horizontal and vertical ladder needs another write-up.

**Table-I:** Layout of themes / modules for integrated medical curriculum*

Year	Modules*								
Year 5	Treatment VI (Mother n Baby / Neurological medicine)			Research methods II		Pre specialization		Entrepreneurship	
Year 4	Treatment III (Community Health / Special Senses)			Research methods I		Treatment IV (Injury / Trauma)		Treatment V (Medical Emergency)	
Year 3	Diagnosis II (Surgical based disciplines)		Diagnosis III (medical based disciplines)			Treatment I (Pain)		Treatment II (Public health issues)	
Year 2	CVS*	Renal	Diagnosis I		Skills Surgical based Simulated Patients	Endo*	Repro*	CNS*	PPD
Year 1	Basic Foundation	Haemopoitic	Musculo- Skeletal		Skills Medical based Simulated Patients)	Respiratory		GIT	PPD*

CVS*= Cardiovascular module, CNS*= Central Nervous module, Repro= Reproductive module,

Endo= Endocrinology, PPD* = professional and personal development

### Organization of themes

Structural layout of themes and their modules are shown in Table-I. For example, diagnosis theme is further divided into diagnosis I (general procedures required for all medical cases) while diagnosis II should relate cognitive, psychomotor and affective domains particular to surgical based disciplines (surgery, orthopedic, obstetrics and gynecology etc.) planted for year 3 (Table-I). The module summary is under multiple subheadings such as: Title, Code, Rationale of the module with learning outcomes, Summary, Contents to be taught, Teaching strategy, Student learning time, Assessment scheme and References (Table-II).

**Table-II:** Presentation of theme (module) information *

NO	Module Summary (MS)**									
1	Name of Course / Module / Year	Module Code					Credit Hours			
2	Pre requisite if any	Name(s) of Academic Staff								
3	Rationale of the module: In this module, students will learn the fundamental human body concepts which would provide a baseline for upcoming modules.									
4	Summary of the module: This module deals with features of fundamental body tissues, their functions and interactions with each other. Fundamental tissues such as bones, epithelia connective tissue would be taught and practiced.									
5	Module Learning outcomes (MLO): At the end of this module , students would be able to MLO1: explain basic concept of body tissues, identifying microscopic features and correlating their functions orally. MLO2: describe the haemostasis functions comprehensively correlating the functions and structural components involved. MLO3: express their ideas coherently and logically on fundamental tissues and their biochemical reactions when working independently and cooperatively in both practical sessions and through assignments.									
6	Mapping of Module learning Outcomes (MLO) with Programme Learning Outcomes (PLO)									
	MLO / PLO	PLO 1 Knowledge	PLO 2 Skill	PLO 3 (Critical thinking)	PLO 4 Communication	PLO 5 Team work	PLO 6 Ethics	PLO 7 Lifelong learning	PLO 8 Business	PLO 9 leadership
	MLO 1	√	√		√					
	MLO 2	√		√						
	MLO 3	√		√	√	√				
7	Mode of Delivery: Lecture, Practical , Tutorial , Seminar, Case base study etc									
8	Content Outline (let’s suppose) Lectures = 20 Hours •Structural component of epithelial tissue •Connective tissue •……. Seminar (14 Hours) •Types of epithelial tissue Practical (8 Hours) •……. •……. Problem Based learning (8 Hours)									
9	Assessment methods •End of module examination •Professional examination									
10	References (recommended books)									

** The template of table is adopted from Management and Science University –Shah Alam, Malaysia.

Teaching methodology could be lecture, seminar, tutorial, problem based learning, case base learning, skill labs etc and adopted according to needs of each module. Difficult concepts of topics are placed as lectures, while seminars cover the easiest section of topics. The practices and application oriented sections of texts are to be in tutorials, problem based and case based sessions.

### Assessment

Formative (40%) and summative (60%) assessment formula is for integrated medical curriculum to evaluate the teaching and learning process. For example the formative assessment, for medical emergency module makes an aggregate of 40 percent and it could be through written test, Objective structured clinical examination (OSCE) and log book. The sequence of formative and summative assessments is managed in this way. First assessment described as the formative assessment is done when 30-40% module contents are taught. When 70-80 % course contents are taught, second (formative) assessment should be placed and a relatively longer assessment, termed as summative assessment is carried out after completing the whole module. The formative assessment strategies could be chosen by medical educators with the help of faculty at their own choice (Table-III). Table-IV is about credit hours. For an undergraduate five years medical program, two hundred to two hundred and fifty credit hours are suitable. However, this debate needs to be reviewed and evaluated because learning process varies among individuals and groups.

**Table-III:** Formative and Summative assessments methods.

Type of assessment	Assessment method	Percentage
Formative Assessment	Mid module examination	
	End of module examination	
	Objective Structured Practical Examination (OSPE)	
	Objective Structured Clinical Examination (OSCE)	
	Viva voce	
	Assignment	
	Log book	
	Clinical case write up	
	Clinical lab report	
	Attendance	
Total Formative Assessment	------------	40
Total Summative Assessment	Professional Exam at end of Year	60

**Table-IV:** Calculation of Credit hour* (an analogue of learning a car driving)

No	Driving school (Face to Face)	Independent learning (Non Face to Face)	Exam (30 minutes)	Result	Credit Hours
First Time	Lecture (2 Hours)	4 Hours	½ Hour	Passed (Theory)	2 + 4 + 0.5 = 6.5 Hrs
	Practical training (20 Hours)	------	½ Hour	Failed (Practical)	20 + 0.5 = 20.5 Hrs
Second Time	------	He does practice of driving by himself. He practiced 60 Hours	½ Hour	Passed and awarded driving license	60 + 0.5 = 60.5 Hrs
Total teaching and learning hours Credit Hours					6.5 + 20.5 + 60.5 = 87.5 87.5 /40 = 2.1

* Learning outcome is *driving* achieved by lecture + practical skills (Face to Face) + Non Face to Face(self-study and self-practice) which are calculated (87.5 Hours) and divided by 40 - a full time working time of a week (employee or full time student). Assessment is via *theory and practical (driving) exam* and this learning outcome (driving) is a 2 credit hour course.

## DISCUSSION

Integrated curriculum provides learners to invest time on leaning a topic only after they understand its importance which is based on the core value of the theme.^14^ Each segment is taught in distinct scale starting from normal to disordered tissue followed by the treatment section. In traditional curriculum, there is either very less or absolute null connection of clinical sciences with basic sciences. This gap has been overcome by joining basic sciences with clinical sciences through problem based, case based or patient based learning in an integrated curriculum, Knowledge is more effective when delivered in an organized form.^15^ A long term retention and deeper understanding can be achieved once connections are made of basic sciences with clinical examples.^16^ Bloom et al.,^17^ described learning having three domains: cognitive, psychomotor and affective which are well-tailored to current format of the curriculum. Moreover, affective domain is further expanded along teaching and learning. A major challenge is to implement an integrated curriculum with various teaching approaches. United faculty as one team rather than divided such as preclinical or clinical faculty, could solve the challenge. Thus a problem based learning (PBL) teaching and learning activity involves faculty members regardless they are subject expert or non-subject oriented. Integrated medical curriculum demands various teaching methodologies. Lecture, seminar, tutorial, problem based learning, case based learning, bedside teaching and clerkship are different approaches for teaching and learning activities.

In recent years, research component has been integrated into the undergraduate medical curriculum and it has become an emerging trend. The benefits of research component include teamwork experience, enhanced presentation skills, applied research with industrial collaboration and deepening critical thinking. It greatly enhances their ability to pursue towards new knowledge while exploring with interest and concentration in a desired area.^18^Entrepreneurship has a significant positive effect on the rate of economic growth.^19^ The entrepreneurship module is to run by inviting faculty of business school or hiring qualified persons. Medical professionalism and ethics are important themes in the medical curriculum. Most of the countries have established their own national medical regulatory bodies which monitor ethical issues in the medical profession.^20^This is a social and professional norm that distinguishes between right and wrong actions and behaviors.^21^ It is an intrinsic part of medical practice and gives responsibilities to health care providers and patients in different circumstances. This theme provides information to the students that would protect them in their practical life. However, no quantitative or qualitative literature is available on this aspect yet.

Pre-specialization is a new concept and none of the medical curriculum has included this theme. This module provides an opportunity for undergraduate students to streamline their post-graduate plans during their under-graduate degree programme. For this purpose, we suggest to invest 4-6 weeks in the final year of the medical programme.

In the suggested themes of integration, basic sciences are assessed by adding clinical reasoning and skills to provide valuable evidence for the effectiveness of certain integration strategy.^22^ In the proposed template of assessment, multiple options are recommended and each school and faculty are free to choose assessment methods for their learners.

### Limitations of the study

How much the theme based integration is effective in producing future physicians who are knowledgeable, practically sound and have high moral values and uphold professional ethics. and since this has not been implemented yet, its critical evaluation is one of the limitations of this study.

## CONCLUSIONS

Contents of traditional disciplines are assorted, harmonized and mutated in particular theme and subsequently distributed into modules. Imparting one whole traditional discipline is not advised into one module; rather, it should be segmented into various parts. The depicted approach is one of many approaches for designing and developing integrated medical curriculum. The integrated curriculum emphasizes on student centered rather than teacher centered learning. Medical schools may choose the different strategies according to their infrastructure and strength of human resources and fulfil the needs of future demands of physicians in the society.

## Availability of Data and Materials

Data is not publicly available as it may comprise information about individual contributors.

## References

[1] David GB, Kristi JF (2015). The integrated curriculum in medical education:AMEE Guide No.96. Med Teach.

[2] Cook M, Irby DM, Sullivan W, Ludmerer KM (2006). American medical education 100 years after the Flexner report. N Engl J Med.

[3] Irby DM, Cooke M, O'Brien BC (2010). Calls for reform of medical education by the Carnegie Foundation for the Advancement of Teaching:1910 and 2010. Acad Med.

[4] Finnerty EP, Chauvin S, Bonaminio G, Andrews M, Carroll RG, Pangaro LN (2010). Flexner revisited:The role and value of the basic sciences in medical education. Acad Med.

[5] Maeshiro R, Johnson I, Koo D, Parboosingh J, Carney JK, Gesundheit N (2010). Medical education for a healthier population:Reflection on the Flexner report from a public health perspective. Acad Med.

[6] Harden RM, Sowden S, Dunn WR (1984). Educational strategies in curriculum development:the SPICES model. Med Educ.

[7] (2001). The Association of American Medical Colleges (AAMC).Report IV- Contemporary issues in medicine:Basic science and clinical research. Medical school objectives project.

[8] (2010). General Medical Council (GMC). Update:Standards for curricula and assessment systems.

[9] (2012). Australian Medical Council Limited. Standards for assessment and accreditation of primary medical programs by the Australian Medical Council 2012. Kingston ACT:AMC.

[10] Christopher RD, Edward C T, Anthony SB, Mathew DC, Frank C, Smith T (2014). Surgical and procedural skills at medical school –a national review. Int J Surg.

[11] Oliver CM, Hunter SA, Ikeda T, Galletly DC (2013). Junior doctor skill in the art of physical examination:a retrospective study of the medical admission note over four decades. BMJ Open.

[12] Joseph B Meleca, Maria Tecos, Abigail L Wenzlick, Rebecca Henry, Patricia A Brewer (2014). A medical student initiated elective course in business and finance:a needs analysis and pilot. Med Stud Res J.

[13] Nicola B, Kate LM, Tim R, Mwapatsa M, Adamson SM (2012). Postgraduate career intentions of medical students and recent graduates in Malawi:a qualitative interview study. BMC Med Educ.

[14] Kaufman DM, Mann KV, Swansick T Teaching and learning in medical education:How theory can inform practice. Understanding medical education:Evidence, theory and practice.

[15] Ambrose SA, Bridges MW, DiPietro M, Lovett MC, Norman MK (2010). How learning works:Seven research based principles for smart teaching.

[16] (2000). National Research Council. How people learn. Brain, mind, experience and school:Expanded edition.

[17] Bloom BS, Engelhart MD, Furst EJ, Hill WH, Kratwohl DR (1956). Taxonomy of educational objectives:The classification of educational goals. Handbook I, Cognitive Domian.

[18] Laidlaw A, Aiton J, Struthers J, Guild S (2012). Developing research skills in medical students AMEE guide no 69. Med Teach.

[19] Bednarzick RW (2000). The role of entrepreneurship in U.S. and European job growth. Monthly Labor Rev.

[20] Beauchamp TL, Childress JF (2001). Principles of biomedical ethics.

[21] Christine G, Marion D, Karen LS, Patricia D, Carol T, Adrienne F, Connie MU (2008). Does ethics education influence the moral action of practicing nurses and social workers?. Am J Bioeth.

[22] Kulasegaram KM, Martimianakis MA, Mylopoulos M, Whitehead CR, Woods NN (2013). Cognition before curriculum:Rethinking the integration of basic science and clinical learning. Acad Med.

